# Effect of hypoproteinemia on the mortality of sepsis patients in the ICU: a retrospective cohort study

**DOI:** 10.1038/s41598-021-03865-w

**Published:** 2021-12-21

**Authors:** Jing Hu, Chenwei Lv, Xingxing Hu, Jiangyun Liu

**Affiliations:** 1 Nanjing University of Traditional Chinese Medicine, Nanjing, Jiangsu China; 2 Affiliated Hospital of Integrated Traditional Chinese and Western Medicine, Nanjing University of Chinese Medicine, Nanjing, China; 3 Jiangsu Province Academy of Traditional Chinese Medicine, Nanjing, China; 4 Emergency Department, Jiangsu Province Academy of Traditional Chinese Medicine, No.100 Cross Street, Hongshan Road, Nanjing, Jiangsu China; 5 Neurology Department, Jiangsu Province Academy of Traditional Chinese Medicine, No.100 Cross Street, Hongshan Road, Nanjing, Jiangsu China

**Keywords:** Diseases, Infectious diseases

## Abstract

The objective of the study was to evaluate the effect of hypoproteinemia on the prognosis of sepsis patients and the effectiveness of exogenous albumin supplementation. A retrospective cohort study was conducted in adult ICUs. The subjects were 1055 sepsis patients in MIMIC III database from June 2001 to October 2012. There were no interventions. A total of 1055 sepsis patients were enrolled and allocated into two groups based on the lowest in-hospital albumin level: 924 patients were in the hypoproteinemia group (the lowest in-hospital albumin ≤ 3.1 g/dL) and 131 patients were in the normal group (the lowest in-hospital albumin > 3.1 g/dL). A total of 378 patients [331 (35.8%) were in the hypoproteinemia group, and 47 (35.9%) were in the normal group] died at 28 days, and no statistically significant difference was found between the two groups (P = 0.99). The survival analysis of the 28-day mortality rate was performed using the Cox proportional risk model and it was found that the lowest in-hospital albumin level showed no significant effect on the 28-day mortality rate (P = 0.18, 95%CI). Patients in the hypoproteinemia group exhibited a longer length of stay in ICU and hospital and more complications with AKI than those in the normal group. However, multivariate regression analysis found that there was no statistical significance between the two groups. In addition, multivariate regression analysis showed that patients in the hypoproteinemia group had a shorter time without vasoactive drugs and time without mechanical ventilation than those in the normal group (P < 0.01). In the subgroup analysis, univariate analysis and multivariate regression analysis showed that there was no significant difference in the 28-day mortality rate (39.6% vs 37.5%, P = 0.80), the proportion of mechanical ventilation time (P = 0.57), and vasoactive drug time (P = 0.89) between patients with and without albumin supplementation. However, patients in the albumin supplementation group had a longer length of ICU stay and hospital stay than those in the non-supplementation group (P < 0.01). Albumin level may be an indicator of sepsis severity, but hypoproteinemia has no significant effect on the mortality of sepsis patients. Despite various physiological effects of albumin, the benefits of albumin supplementation in sepsis patients need to be evaluated with caution.

## Introduction

Sepsis is one of the diseases with high morbidity and fatality rate in ICU worldly, and the current fatality rate remains as high as 25–30%^1^. Due to protein-rich fluid extravasation^2^ caused by capillary dysfunction and other factors, hypoalbuminemia occurs more frequently in sepsis patients, especially in those septic shock patients. Previous studies have shown that the decreased serum albumin level is one of the risk factors for the increased mortality in sepsis^3,4^. However, this concept is still controversial^5^, especially when albumin is used as resuscitation fluid. Although albumin infusion is safe, it does not reduce the fatality rate^6^. Based on data from the MIMIC III database, this study aims to further clarify the effect of hypoproteinemia on the prognosis of sepsis patients and the necessity of exogenous albumin supplementation.

## Materials and methods

### Database introduction

The Medical Information Mart for Intensive Care III (MIMIC-III) dataset is a large, de-identified and publicly-available collection of medical records associated with over forty thousand patients who stayed in critical care units of the Beth Israel Deaconess Medical Center in Boston between 2001 and 2012. A total of 61,532 (53,432 adult patients and 8100 neonatal patients) intensive care unit inpatients were included. The data in this study was extracted using Postgre SQL 9.6 software. One of the researchers, Chengwei Lv, has completed the online training course of the National Institutes of Health (certification number: 39524425). The regional Ethical Review Board of Laboratory for Computational Physiology at the Massachusetts Institute of Technology approved the study. All the patients in the database were de-identified for privacy protection, and the need for informed consent was waived. The link of this database is https://mimic.mit.edu/.

### Inclusion and exclusion criteria

In this study, sepsis patients who have the admitted albumin levels were recruited from MIMIC III. The inclusion criteria were: (1) age ≥ 18 years; and (2) diagnosis of sepsis, severe sepsis, or septic shock in MIMIC III database. The exclusion criteria were: (1) age < 18 years; (2) patients in puerperium; (3) low serum protein caused by wasting diseases (such as tumor, connective tissue disease, and hematological disease); and (4) no recorded albumin level.

### Data extraction

Data extraction was performed in Postgre SQL (v9.6) using Structured Query Language (SQL). The following information was extracted: age, gender, BMI, comorbidities [including severe sepsis, septic shock, chronic obstructive pulmonary disease (COPD), coronary heart disease, hypertension, heart failure, cerebrovascular accident, chronic kidney disease, and diabetes], Sequential Organ Failure Assessment (SOFA) score, Simplified Acute Physiology Score II (SAPS II) score, and laboratory examination on admission (such as examination for white blood cells, hemoglobin, platelets, blood sodium, blood potassium, blood sugar, blood lactic acid, blood creatinine, serum albumin, the lowest in-hospital albumin level, and the use and duration of vasoactive drugs and mechanical ventilation).

### Study endpoint

The primary study endpoint was 28-day all-cause mortality. The secondary study endpoints were the length of ICU stay, length of hospital stay, duration of non-vasoactive drug use, and no mechanical ventilation time. The 28-day all-cause mortality was defined as death resulting from any cause within 28 days after ICU hospitalization.

### Statistical analysis

The categorical variables were expressed as percentages and compared using the Chi-square test. The continuous variables were represented as mean ± standard deviation or median (interquartile range), and analyzed using the T test or rank-sum test. The rough relationship between the lowest in-hospital albumin level and the 28-day mortality rate was detected using the LOWESS technique. The lowest in-hospital serum albumin at the lowest point of the 28-day mortality rate was determined as 3.1 g/dL, on which patients were grouped (the hypoproteinemia group with the lowest in-hospital albumin ≤ 3.1 g/dL and the normal group with the lowest in-hospital serum albumin > 3.1 g/dL). The survival curve of different albumin levels was established by Kaplan–Meier (K–M) analysis, and the log-rank test was used for comparison. The multivariate logistic regression was used for further analysis of the relationship between albumin levels and the 28-day mortality rate, and a reverse stepwise regression filter was adopted for covariates. Subgroup analysis was performed based on albumin supplementation. Data missing values were filled using the median, mode, and regression filling method according to the specific situation. The significance level was set as 0.05. STATA 15.0 software was used for analysis.

### Human subjects

All studies were performed using ethical principles for medical research involving human subject in accord with the Declaration of Helsinki.

### Ethical approval and consent to participate

Laboratory for Computational Physiology at the Massachusetts Institute of Technology. In addition, all the patients in the database were de-identified for privacy protection, and the need for informed consent was waived, so it is not required an approval by an ethical committee or a regulatory authority of China (local or national).

### Consent for publication

The Author confirms: (1) that the work described has not been published before (except in the form of an abstract or as part of a published lecture, review, or thesis); (2) that it is not under consideration for publication elsewhere; (3) that its publication has been approved by all coauthors, if any; (4) that its publication has been approved (tacitly or explicitly) by the responsible authorities at the institution where the work is carried out.

## Results

### Population and baseline characteristics

**Figure 1:**
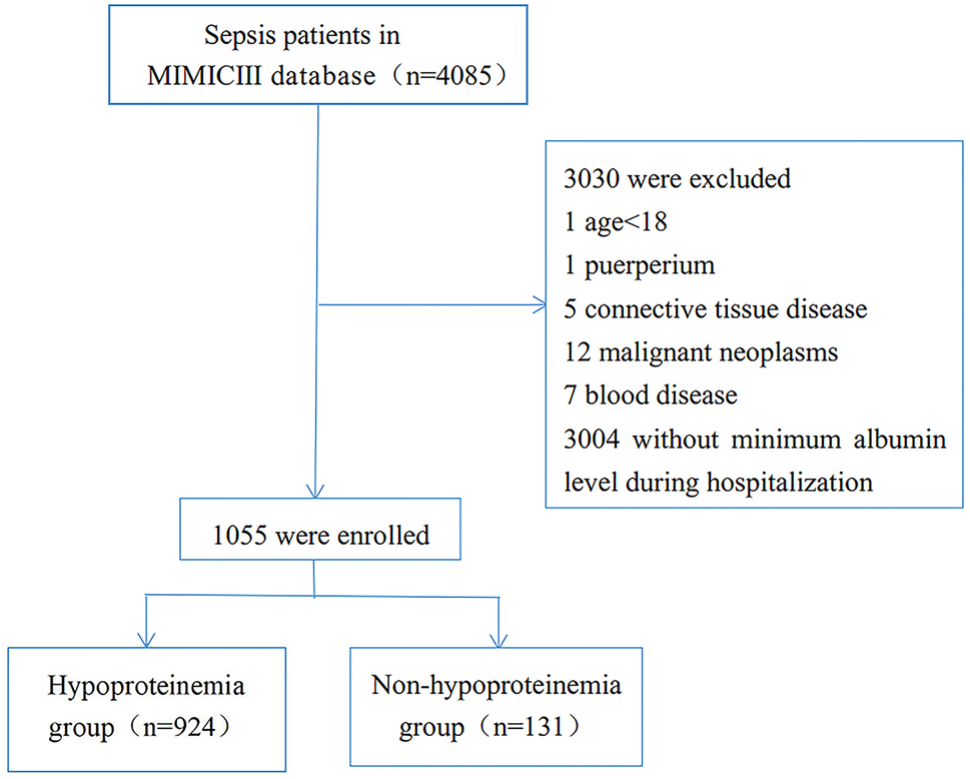
Flow chart.

A total of 4085 sepsis admissions were recorded in the MIMIC III database, of which 3030 cases were excluded [age < 18 years (1 case), puerperium (1 case), connective tissue disease (5 cases), hematological disease (7 cases), malignant tumor (12 cases), and no recorded serum albumin level during hospitalization (3004 cases)]. The specific inclusion process is shown in Fig. 1. Finally, 1055 hospitalized patients [600 males (56.8%), median age of 67.0 (55.0–79.3) years] were included in this study. The median SOFA score was 8 points (5–11). The most common baseline diseases were septic shock (70.7%), diabetes (352, 33.3%), hypertension (427, 30.9%), and heart failure (30.1%).

**Figure 2:**
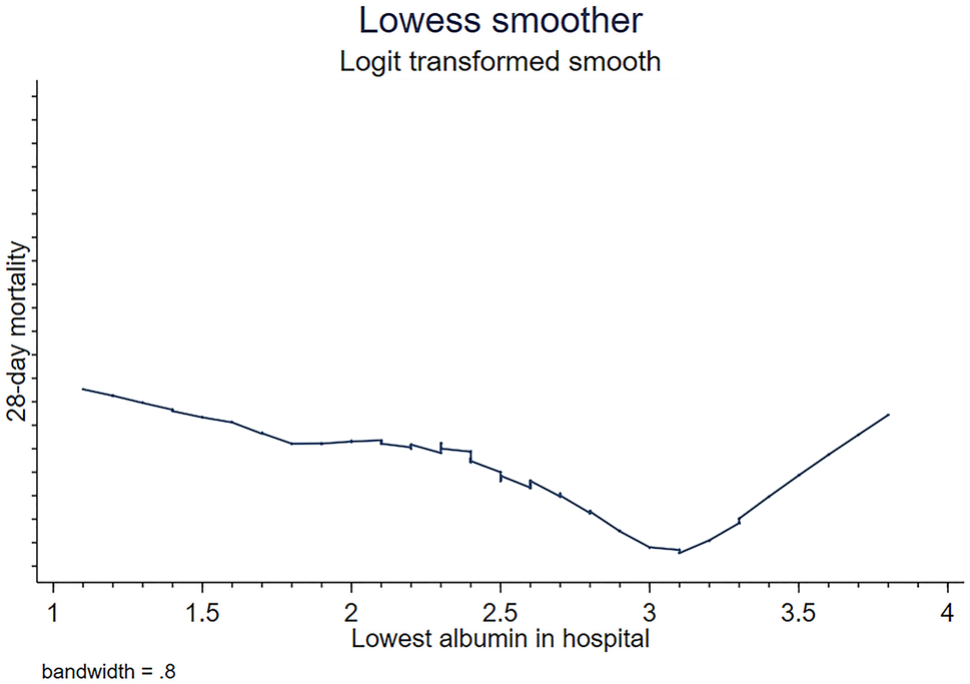
The Lowess smoothing technique shows the relationship between the lowest in-patient serum albumin level and the 28-day mortality rate.

The LOWESS technique was used to examine the rough relationship between the lowest in-hospital albumin level and the 28-day mortality rate (Fig. 2). The lowest in-hospital albumin level at the lowest point of 28-day mortality was determined as 3.1 g/dL, on which the patients were allocated into the hypoproteinemia group (924 cases, the lowest in-hospital albumin level ≤ 3.1 g/dL) and the normal protein group (131 cases, the lowest in-hospital albumin level > 3.1 g/dL). The SAPSII score of the two groups was significantly different [8 (5–11), 7 (4–10), P < 0.01]. Patients in the hypoalbuminemia group had significantly lower serum albumin level at admission than those in the normal group [2.5 (2.1–2.9) vs 3.3 (3.2–3.7), P < 0.01], along with the lowest in-hospital albumin level [2.2 (1.9–2.6) vs 3.3 (3.1–3.5), P < 0.01]. Moreover, the number of septic shock patients (P < 0.03) and chronic kidney patients (P < 0.03), the level of platelets at admission (P < 0.02), and the number of vasoactive drugs (P < 0.01) and mechanical ventilation (P < 0.01) were also significantly different in the two group. In addition to these, other baseline characteristics showed no difference between patients in the two groups. The demographic characteristics of the two groups are shown in Table 1**.**

**Table 1:** Baseline situation of the study population.

Variable	Total (n = 1055)	Hypoproteinemia group (n = 924)	Normal group (n = 131)	P
Age, median (IQR)	67.0 (55.0–79.3)	67.1 (55.8–79.3)	64.3 (49.9–80.3)	0.22
Male, n (%)	600 (56.8)	520 (56.2)	80 (61.0)	0.30
BMI, median (IQR)	28 (24.1–32.8)	27.9 (24.0–32.7)	28.7(24.5–33)	0.89
SOFA score, median (IQR)	8 (5–11)	8 (5–11)	7 (4–10)	0.23
SAPSII score, median (IQR)	47 (36–58)	48 (37–59)	43 (31–54)	< 0.01
**Sepsis, n (%)**				
Septic shock	746 (70.7)	664 (71.8)	82 (62.5)	0.03
Severe sepsis	1053 (99.8)	922 (99.7)	131 (100)	0.59
**Comorbidities, n (%)**				
COPD	22 (2.0)	18 (1.9)	4 (3.0)	0.41
Coronary heart disease	211 (20)	182 (19.6)	29 (22.1)	0.51
Hypertension	427 (30.9)	376 (40.6)	51 (38.9)	0.70
Heart failure	318 (30.1)	275 (29.7)	43 (32.8)	0.48
Cerebrovascular accident	4 (0.3)	3 (0.3)	1 (0.7)	0.45
Chronic kidney disease	215 (20.3)	179 (19.3)	36 (27.4)	0.03
Diabetes	352 (33.3)	300 (32.4)	52 (39.6)	0.10
**Laboratory examination on admission, median (IQR)**				
White blood cells (10^9^/L)	12.9 (7.6–18.5)	13 (7.6–18.8)	12.1 (7.6–17.5)	0.21
Hemoglobin (g/dL)	10.2 (9–11.8)	10.2 (9–11.6)	10.8 (9.2–12.3)	0.07
Platelets (10^9^/L)	183 (114–269)	187 (116–276)	173 (101–222)	< 0.01
Blood potassium (mmol/L))	4 (3.6–4.5)	4 (3.6–4.5)	4 (3.6–4.5)	0.88
Blood sodium (mmol/L)	138 (134–141)	138 (134–141)	138 (135–141)	0.89
Blood sugar, (mg/dL)	129 (102–168)	128 (101–168)	137 (111–180)	0.21
Serum albumin (g/dL)	2.6 (2.2–3)	2.5 (2.1–2.9)	3.3 (3.2–3.7)	< 0.01
Blood lactic acid (mmol/L)	2 (1.3–3.1)	2 (1.3–3.1)	1.8 (1.4–3.1)	0.87
Blood creatinine (mg/dL)	1.4 (0.9–2.4)	1.4 (0.9–2.4)	1.6 (1–3)	0.03
The lowest in-hospital albumin level (g/dL), median (IQR)	2.3 (2–2.8)	2.2 (1.9–2.6)	3.3 (3.1–3.5)	< 0.01
Albumin supplementatio, n (%)	350 (33.1%)	318 (34.4%)	32 (24.4%)	0.02
The use of vasoactive drugs, n (%)	801 (75.9%)	722 (78.1%)	79 (60.3%)	< 0.01
The use of mechanical ventilation), n (%)	632 (59.9%)	567 (61.3%)	65 (49.6%)	0.01

### Primary and secondary study endpoints

**Figure 3:**
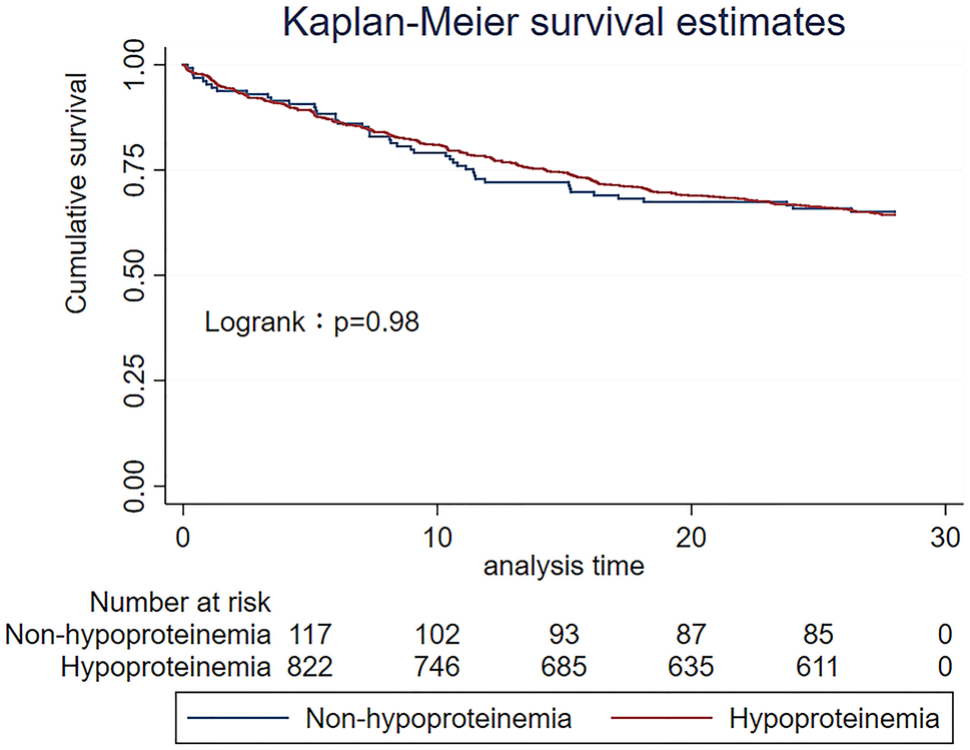
The 28-day mortality K-M curve of the albumin group.

For the primary endpoint, 378 patients [(331 (35.8%) in the hypoproteinemia group and 47 (35.8%) in the normal group)] died at 28 days, with no statistical difference (P = 0.99), which was further verified by multivariate regression analysis (P = 0.18). To further explore the relationship between serum protein level and the 28-day mortality rate of patients, we performed a survival analysis of the Cox proportional hazard model on the 28-day mortality rate and the K-M curve was plotted (Fig. 3). The results showed that SAPSII score, diabetes mellitus, and initial lactate level had a notable impact on the 28-day mortality rate. Indicators (such as the initial albumin level on admission and the lowest in-hospital albumin level) showed no significant impact on the 28-day mortality rate. The data is shown in Table 2. The survival analysis revealed that the lowest in-hospital albumin level exerted no significant effect on the 28-day mortality rate (P = 0.18, 95% CI) (see Fig. 3).

**Table 2:** Regression analysis of multivariate Cox ratio of the 28-day mortality rate at the lowest serum protein level.

	Haz. ratio	Std. err.	z	P > |z|	[95% conf. interval]
The lowest in-hospital albumin level	0.75	0.15	− 1.33	0.18	0.49–1.14
SAPSII score	1.03	0.003	11.23	< 0.01	1.03–1.04
Chronic kidney disease	0.98	0.13	− 0.12	0.91	0.74–1.29
Diabetes mellitus	0.77	0.09	− 2.15	0.03	0.62–0.97
Septic shock	1.11	0.13	0.88	0.38	0.87–1.40
**Laboratory examination on admission**					
Platelets	0.99	0.00	− 0.64	0.52	0.99–1.00
Lactate level	1.11	0.02	5.16	< 0.01	1.06–1.16
Albumin level	0.79	0.11	− 1.57	0.12	0.60–1.05
Blood creatinine	0.95	0.03	− 1.12	0.26	0.89–1.03
Hemoglobin	0.97	0.02	− 1.02	0.31	0.92–1.02
The lowest in-hospital albumin level	1.26	0.21	1.34	0.18	0.89–1.76
Albumin supplementation	1.03	0.11	0.29	0.77	0.82–1.29

In the secondary study endpoints, patients in the hypoproteinemia group had longer ICU stay and hospital than those in the normal group. In this aspect, no statistically significant difference was found between the two groups after multivariate regression analysis. However, multivariate regression analysis revealed that patients in the hypoproteinemia group had remarkably longer time without vasoactive drugs and the time without mechanical ventilation than those in the normal group (P < 0.01) (Table 3).

**Table 3:** The effect of the lowest in-hospital albumin level on the clinical outcome of 1055 patients.

Variable	Hypoproteinemia group (n = 924)	Normal group (n = 131)	P univariate	P multivariate
The time without vasoactive drugs	3.6 (1.6–9.3)	2.3 (1.3–5.1)	< 0.01	< 0.01
The time without mechanical ventilation	2.3 (1.2–4.2)	2.0 (1.2–3.5)	0.17	< 0.01
ICU stay	6.0 (2.7–13.0)	3.5 (1.8–7.0)	< 0.01	0.55
Hospital stay	13.7 (7.1–24.1)	8.1 (5.3–12.3)	< 0.01	0.63

In multivariate regression analysis, the influences of factors (such as SAPSII score, septic shock, chronic kidney disease, diabetes, hemoglobin, platelets, serum albumin, the lowest in-hospital albumin level, albumin supplementation, use of vasoactive drugs, and mechanical ventilation) were excluded.

### Subgroup analysis

To observe the relevant effects of exogenous albumin supplementation on the prognosis of patients with hypoalbuminemia and sepsis, we further conducted analysis on patients in the hypoalbuminemia group. The patients were allocated into two groups [group A (with exogenous albumin supplementation) and group B (without exogenous albumin supplementation)] according to whether they were supplemented with exogenous albumin during hospitalization. A caliper value of 0.02 was used for a 1:1 tendency match to reduce the influence of confounding factors (such as albumin level and disease severity) that may cause bias in the results. Variables (SOFA score, initial value of lactic acid, coronary heart disease, diabetes, heart failure, hospitalized hemoglobin, initial value of platelets, initial value of blood potassium, initial value of blood sodium, initial albumin, and the lowest in-hospital albumin level) were selected for propensity scoring. Finally, 280 matching pairs were generated and used for further analysis. The baseline status of patients in the two groups is shown in Table 4.

**Table 4:** Baseline situation of the two groups with and without albumin supplementation after propensity matching analysis.

Variable	Albumin supplementation (A) (n = 280)	No albumin supplementation (B) (n = 280)	P
Age, median (IQR)	65.9 (55.9–78.2)	67.1 (53.8–79.9)	0.97
Male, n (%)	167 (59.6)	156 (55.7)	0.35
BMI, median (IQR)	28.4 (25–32.7)	27.7 (22.7–31.9)	0.04
SOFA score, median (IQR)	8 (5–11)	8 (6–11)	0.58
SAPSII score, median (IQR)	49 (39–58)	50 (37–61)	0.37
**Sepsis, n (%)**			
Septic shock	192 (68.5)	206 (73.5)	0.19
Severe sepsis	280	280	
**Comorbidities, n (%)**			
COPD	4 (1.4)	8 (2.8)	0.24
Coronary heart disease	44 (15.7)	41 (14.6)	0.72
Hypertension	120 (42.8)	107 (38.2)	0.26
Heart failure	71	71	
Cerebrovascular accident	1	1	
Chronic kidney disease	48 (17.1)	57 (20.3)	0.33
Diabetes	86 (30.7)	74 (26.4)	0.26
**Laboratory examination on admission, median (IQR)**			
White blood cells (10^9^/L)	12.3 (7.4–17.6)	12.9 (7.4–19.8)	0.64
Hemoglobin (g/dL)	10.2 (9–11.9)	10.4 (9.1–12)	0.70
Platelets (10^9^/L)	178 (112–268)	167 (100–263)	0.30
Blood potassium (mmol/L)	4.1 (3.6–4.6)	4 (3.6–4.6)	0.67
Blood sodium (mmol/L)	138 (134–142)	137 (133–141)	0.18
Blood sugar, (mg/dL)	127 (99–165)	129 (104–170)	0.49
Serum albumin (g/dL)	2.4 (2–2.8)	2.5 (2.1–2.8)	0.84
Blood lactic acid (mmol/L)	2.1 (1.4–3.4)	2.2 (1.3–3.4)	0.70
Blood creatinine (mg/dL)	1.4 (0.9–2.5)	1.4(0.9–2.4)	0.85
The lowest in-hospital albumin level (g/dL), median (IQR)	2.1 (1.8–2.5)	2.1 (1.9–2.5)	0.71
The use of vasoactive drugs, n (%)	240 (85.7)	215 (76.7)	< 0.01
The use of mechanical ventilation), n (%)	196 (70)	168 (60)	0.01

According to single-factor analysis and multivariate regression analysis, there was no significant difference between patients in group A and B in the 28-day mortality rate (39.6% vs 37.5%, P = 0.80), the proportion of mechanical ventilation time (P = 0.57), and the time of vasoactive drugs (P = 0.89). Nevertheless, patients in group A had a notably longer length of ICU stay and hospital stay than those in group B (P < 0.01) (Table 5).

**Table 5:** The effect of albumin supplementation for hypoalbuminemia on the results of the study.

Variable	Albumin supplementation (A) (n = 280)	No albumin supplementation (B) (n = 280)	P univariate	P multivariate
Mechanical ventilation time %	43.1 (7.9–77.8)	32.9 (0–66.6)	0.01	0.57
Vasoactive drugs time, %	18.4 (4.1–40.4)	13.3 (0.7–34.4)	0.03	0.89
AKI, n (%)	217 (80.3)	195 (69.6)	0.04	0.06
ICU stay, day, median (IQR)	10.6 (4.4–18.6)	5.2 (2.6–11.6)	< 0.01	< 0.01
Hospital stay, day, median (IQR)	21.5 (12.8–33.5)	10.9 (5.9–20.3)	< 0.01	< 0.01
28-day mortality rate, n (%)	111 (39.6)	105 (37.5)	0.60	0.80

In multivariate regression analysis, possible influencing factors (including BMI, septic shock, blood sodium on admission, vasoactive drugs, and mechanical ventilation) were excluded.

## Discussion

Albumin, as the most abundant protein in the circulation, accounts for 50% of the total plasma protein, with a normal serum range of 3.5–5.0 g/dL^3^. Serum albumin may be beneficial to reduce thrombosis, with various physiological functions, such as inhibition of platelet aggregation, antioxidantion, maintaining endothelial stability, anti-inflammation, and acting as a carrier for many physiological and pharmacological important molecules^7,8^. Due to the inconsistency of diagnostic criteria, the incidence of hypoalbuminemia in sepsis patients is as high as 70–80%^9^. In this study, the lowest in-hospital serum albumin at the 28-day mortality point of the included cases of 3.1 g/dL was used as the cutoff value for grouping, which is closer to the target albumin level (3.0 g/dL) in the Caironi study^6^.

Arnau-Barrés has found that in elderly sepsis patients with sepsis, the non-survivors admitted to the hospital show a notably lower albumin level than survivors (2.6 vs 3.1 mg/dL, P < 0.01). The lowest albumin level is a potent predictor of mortality, promising to be a biomarker for prognosis and future potential interventions^4^. Seo has found that hypoproteinemia, high BUN, and high CK-MB values are predictive of the increased mortality^10^ in patients with severe sepsis or septic shock in the emergency department. However, albumin levels are affected by nutritional status and chronic inflammation. Therefore, predictions only based on albumin level may have limitations^11^. This study found that patients in the hypoproteinemia group and the normal group showed different initial albumin (2.5 vs 3.3, P < 0.01) and the lowest albumin levels (2.2 vs 3.3, P < 0.01). However, both initial albumin and lowest albumin level had no significant effect on the 28-day mortality rate of sepsis patients in two groups (P 0.12, 0.18). On the contrary, SAPSII score (P < 0.01), diabetes mellitus (P < 0.03), and initial lactate level (P < 0.01) had a significant impact on the 28-day mortality rate. Although hypoalbuminemia seemed to cause more serious damage to organ function (as manifested by the prolonged time of mechanical ventilation and the use of pressure-boosting drugs) in sepsis patients, no statistically significant difference was found compared with patients with high albumin levels. This result is inconsistent with previous studies^3,4^, which may be related to the small number of cases in the previous study and the inconsistency of the study population.

The mechanism of hypoalbuminemia during sepsis may be that: (1) sepsis causes capillary leakage, thus albumin enters the interstitial fluid^12^; (2) sepsis causes a state of high catabolism in patients, and albumin is decomposed^13^; and (3) albumin is synthesized in the liver, and sepsis-caused inflammatory factors lead to the decline of liver synthesis function^14^. Given that albumin function in increasing colloidal osmotic pressure and maintaining blood volume, albumin supplementation seems to be beneficial for sepsis patients with hypoalbuminemia. However, in a previous study, Caironi supplements albumin to sepsis patients to correct hypoalbuminemia, which does not improve the 28-day and 90-day mortality rates^6^. In the subgroup analysis in this study, patients in the hypoproteinemia group were subgrouped based on albumin supplementation, and propensity matching was performed according to disease severity and the albumin level to ensure that the two groups of patients were worthy of comparison. Although we don't know the purpose, method, and total dose of albumin supplementation by clinicians, there was no significant difference in the 28-day mortality rate between groups with and without albumin supplementation. However, patients with albumin supplementation had a longer length of ICU stay and hospital stay than those without albumin supplementation (all P < 0.01). For this reason, we need to be more cautious about exogenous protein supplementation although there was no significant difference in the ratio of mechanical ventilation time and vasoactive drug time in two groups of patients.

Under the background of COVID-19 pandemic, Amira Mohammed Al’s study reminds clinicians to pay attention to hypoproteinemia in COVID-19 patients, which may be one of the predictors of poor prognosis^15^. The studies included in this review are mostly patients with severe COVID-19, and the case fatality rate can be as high as 49%^16^. Among these patients, 59% of patients have liver damage, which is the cause of poor prognosis such as increased mortality^17^. The albumin is synthesized in the liver, which makes the reasons for the reduction of albumin more complicated. Whether it can be used as a predictor of mortality requires more large-scale studies to confirm. However, similar to our study, Amira Mohammed Al’s study also shows that hypoproteinemia can be used as one of the evaluation indicators of disease severity.

There are some shortcomings in this study: (1) the patient's previous nutritional level cannot be obtained due to database limitations, thus the degree of albumin decline caused by this infection cannot be obtained, which may better reflect the severity of the patient's condition; and (2) due to the lack of a unified diagnosis and treatment standard, the concentration, dosage, and treatment timing of albumin supplementation are not recorded in the subgroup analysis. Therefore, there is a certain selection bias in the relationship between albumin supplementation and secondary observation indicators, which need to be further validated. In this study, correcting hypoproteinemia did not change the mortality rate, which is consistent with the research of Caironi.

## Conclusion

Albumin blood level may be one of the indicators for evaluating sepsis severity. However, hypoalbuminemia exerts no significant effect on mortality. Although albumin has various physiological effects, the benefits of albumin supplementation in sepsis patients need to be carefully evaluated.

## Data Availability

The data sets supporting the results of this article are included within the article and its additional files.
